# New technologies in the mix: Assessing N‐mixture models for abundance estimation using automated detection data from drone surveys

**DOI:** 10.1002/ece3.6522

**Published:** 2020-06-30

**Authors:** Evangeline Corcoran, Simon Denman, Grant Hamilton

**Affiliations:** ^1^ School of Earth, Environmental and Biological Sciences Queensland University of Technology (QUT) Brisbane QLD Australia; ^2^ School of Electrical Engineering and Computer Science Queensland University of Technology (QUT) Brisbane QLD Australia

**Keywords:** abundance estimation, hierarchical models, koala, linear models, machine learning, thermal imaging, unmanned aerial vehicles, wildlife detection

## Abstract

1. Reliable estimates of abundance are critical in effectively managing threatened species, but the feasibility of integrating data from wildlife surveys completed using advanced technologies such as remotely piloted aircraft systems (RPAS) and machine learning into abundance estimation methods such as N‐mixture modeling is largely unknown due to the unique sources of detection errors associated with these technologies.
2. We evaluated two modeling approaches for estimating the abundance of koalas detected automatically in RPAS imagery: (a) a generalized N‐mixture model and (b) a modified Horvitz–Thompson (H‐T) estimator method combining generalized linear models and generalized additive models for overall probability of detection, false detection, and duplicate detection. The final estimates from each model were compared to the true number of koalas present as determined by telemetry‐assisted ground surveys.
3. The modified H‐T estimator approach performed best, with the true count of koalas captured within the 95% confidence intervals around the abundance estimates in all 4 surveys in the testing dataset (*n* = 138 detected objects), a particularly strong result given the difficulty in attaining accuracy found with previous methods.
4. The results suggested that N‐mixture models in their current form may not be the most appropriate approach to estimating the abundance of wildlife detected in RPAS surveys with automated detection, and accurate estimates could be made with approaches that account for spurious detections.

## Graphical abstract

We assessed N mixture models to estimate abundance of koalas from data obtained from drone surveillance and machine‐learning algorithms. N mixture models performed poorly due to the false and duplicate detection inherent machine‐learning approaches. An alternative model proposed in the paper worked very well to estimate abundance.

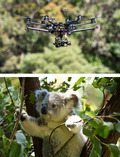

## 1 INTRODUCTION

Abundance data allow monitoring of population trends that can be used to determine management priorities and inform implementation of interventions for the conservation of threatened species (Anderson, 2001; Renwick et al., 2011). While surveys to obtain direct counts are widely used to obtain abundance data (Anderson, 2001), every form of wildlife survey has its own source of errors that may lead to error in abundance estimates (Hone, 2008). These errors include inability to perfectly detect individuals, as well as false detection when other animals or objects are mistaken for the target species (Mackenzie, 2005). Threatened species are often elusive, widely dispersed, and commonly occur in complex environments or inhabit inaccessible areas which increase the potential for imperfect detection and false detections during counts (Hone, 2008). Therefore, statistical modeling approaches that can account for these errors are critical to achieving reliable and informative estimates of abundance for vulnerable species (Mackenzie, 2005; Sollmann et al., 2013).

The modeling methods that have shown to be the most effective in accounting for imperfect detection are methods that require repeated visits to survey sites over time (Ficetola et al., 2018). This includes capture–mark–recapture, removal sampling, and N‐mixture modeling approaches. Although there is debate around which approach is most accurate, evidence has been provided supporting the reliability of each method for estimating abundance, predicting trends in populations over time, and highlighting key factors that influence individual survival and population declines (Barker, Schofield, Link, & Sauer, 2018; Chao, 2001; Link, Schofield, Barker, & Sauer 2018; Petranka & Murray, 2001; Royle, 2004). For cryptic, widely dispersed species, N‐mixture models in which repeated counts at survey sites are used to estimate both probability of detection and population size are often considered the most useful and practical (Ficetola et al., 2018; Royle, 2004). This is because they are the only repeat visit approach that allows estimation of abundance without needing to physically mark individuals, a task that is challenging and costly for elusive species particularly if they occur in private or inaccessible areas (Royle, 2004). Therefore, N‐mixture modeling is less time, cost‐ and labor‐intensive than other repeat visit approaches, estimates can be made over larger areas and the approach is suitable for protected species (Kery et al., 2009).

Some potential issues in applying N‐mixture modeling to wildlife abundance have been highlighted such as a lack of identifiable parameters when abundance, probability of detection, or the number of visits to survey sites is low (Kery, 2017). Additionally, although N‐mixture approaches have been shown to be effective for count data from several common survey methods including point counts, plots, and transects, it is currently unknown whether they can be applied to data gathered from emerging survey methods and technologies (Brack, Kindel, & Oliveira, 2018). For example, there has been increasing interested in applying N‐mixture modeling techniques to data from remotely piloted aircraft systems (RPAS), or drone, are surveys of wildlife (Brack et al., 2018; Kellenberger, Marcos, & Tuia, 2018). This is because RPAS surveys are often employed for species that are difficult to access or easily disturbed by researchers on the ground and therefore are more practical to survey through methods that do not require individuals to be marked (Anderson & Gaston, 2013; Christie, Gilbert, Brown, Hatfield, & Hanson, 2016; Hollings et al., 2018; Kellenberger et al., 2018). Drone data have also recently been used with automated wildlife detection techniques rather than manual identification to reduce observer bias and save time completing surveys and analysis (Chretien, Theau, & Menard, 2016; Corcoran, Denman, Hanger, Wilson, & Hamilton, 2019; Hodgson, Baylis, Mott, Herrod, & Clarke, 2016; Seymour, Dale, Hammill, Halpin, & Johnston, 2017). However, despite the potential advantages of using these new technologies to survey cryptic, widely dispersed and inaccessible species, the unique sources of error that each introduces have not previously been considered in N‐mixture modeling approaches (Baxter & Hamilton, 2018). In particular, the increased potential of spurious detections inherent in using these new survey technologies has not been evaluated as N‐mixture models of abundance assume low probability of detection will be the greatest source of error in surveys and that the effect of false and duplicate detections on counts will be negligible (Ficetola et al., 2018; Royle, 2004).

False and duplicate detections may prove to be more problematic for surveys conducted with RPAS and automated detection than has previously been considered. Spurious detections are prevalent in automated detection studies, particularly in methods that result in a high probability of detection (Brack et al., 2018; Longmore et al., 2017). Recent research demonstrates that when probability of detection reaches 80%, between 3 and 20 false or duplicate detections are likely to be found for each true detection (Kellenberger et al., 2018; Rey, Volpi, Joost, & Tuia, 2017). Additionally, the flight paths (transect lines) in RPAS surveys are often close together to allow the creation of orthomosaic imagery leading to overlap in the field of view (FOV) of sensors between transects of 85% or more. This increases the likelihood of duplicate detections arising from individuals being viewed at different angles (Baxter & Hamilton, 2018; Brack et al., 2018; Denes, Silveira, & Beissinger, 2015; Riddle, Stanislav, Pollock, Moorman, & Perkins, 2010). Previously there have been very limited opportunities to ground truth automated counts of wildlife derived from RPAS imagery, and therefore, it remains an open question whether the aforementioned attributes of these technologies meaningfully impact on the suitability of contemporary N‐mixture models for estimating abundance from the data they provide (Brack et al., 2018; Ficetola et al., 2018; Royle, 2004).

Koalas (*Phascolarctos cinereus*) have been declared threatened throughout the majority of their range and accurate abundance data are needed to inform the management of this species (McAlpine et al., 2006; Scheele et al., 2018). Due to their cryptic nature and wide, patchy distribution conventional ground and aerial surveys of koalas are costly and time‐consuming to conduct and the capacity to identify individuals in repeated visits is typically limited (Adams‐Hosking et al., 2016; Dique, Thompson, Preece, de Villiers, & Carrick, 2003). It has recently been shown that using RPAS and machine learning can result in higher rates of detection for vulnerable koalas than traditional ground survey methods but there remains a margin of uncertainty in these counts that is yet be accounted for in order to accurately estimate abundance (Corcoran et al., 2019). The aim of this paper is to assess the reliability of N‐mixture models for abundance estimation method of wildlife surveyed using RPAS‐derived thermal imagery and automated detection. The abundance of koalas in Petrie, Queensland, in 2018 was estimated using two modeling approaches. A generalized N‐mixture model was developed using the standard approach in the literature (see, e.g., DiRenzio, Che‐Castaldo, Saunders, Grant, & Zipkin, 2019; Kwon et al., 2018). This was compared to an approach using a modified Horvitz–Thompson (H‐T) estimator (Marques, Thomas, Ward, DiMarzio, & Tyack, 2009) incorporating generalized linear models (GLMs) and generalized additive models (GAMs) for overall probability of detection, false detection, and duplicate detection.

## 2 MATERIALS AND METHODS

### 2.1 Survey design and automated detection

A detailed description of the survey design and algorithmic workflow used to generate automated detections for this study are available in Corcoran et al. (2019). In summary, two sites in Petrie, Queensland, were selected for this study as they contained a relatively isolated population of 48 koalas all of which have been fitted with radio collars and tracked extensively by field ecologists, allowing for a rare opportunity to verify when individually identified koalas were detected or missed, and whether individuals were detected multiple times (Hanger, 2017; Waugh et al., 2016). A total of eleven RPAS surveys (six at the south site and five at the north site) were conducted between February and August 2018. RPAS surveys were conducted at first light with a Matrice 600 Pro drone and A3 flight controller (DJI, Shenzhen, China), and a FLIR Tau 2 640 thermal camera (FLIR, Wilsonville, OR, USA) mounted to the underside. A thermal sensor was used because they have been shown to be useful in conjunction with RPAS to increase the probability of detection of cryptic mammals (Hodgson et al., 2016; Seymour et al., 2017). Expert koala trackers conducted ground surveys on the same day as RPAS surveys and the GPS locations of all collared koalas present within the survey site were recorded. The image detection algorithm presented in Corcoran et al. (2019) was then applied to thermal images collected during RPAS and a list of possible koala detections and their GPS co‐ordinates generated. The possible detections were manually examined in order to confirm that the detected koalas matched the co‐ordinates of ground‐surveyed koalas. This allowed verification of the number that had been successfully detected, those that were duplicate detections, the number of false detections, and how many had been missed.

A total of 385 observations from RPAS and group surveys were used in this study. This was split into a training dataset of 247 observations derived from seven surveys (four at the north site and three at the south site) which was used in model development, and a separate dataset comprising a total of 138 detected objects from the four remaining surveys (two at the north site and two at the south site) which was used to test the accuracy of the model at predicting the abundance of koalas from new data. The data were divided this way for two reasons. First, to the best of our knowledge, this site is unique in having such a large number of radio‐collared koalas that are regularly ground surveyed. Population testing data could not therefore be drawn from an entirely separate sampling campaign, and the data from the single campaign at Petrie, Queensland, needed to be divided into distinct groups (Schuwirth et al., 2019; Wenger & Olden, 2012). As many of the covariates explored, in particular ambient temperature and wind speed, are likely to be temporally linked, it seemed most appropriate to divide the data based on survey date (Roberts et al., 2016; Schuwirth et al., 2019; Wenger & Olden, 2012). The four surveys from July 2018 were therefore used in testing and kept separate from the training data to minimize the likelihood of the model relying on temporal autocorrelation to make accurate predictions (Harris, 2015; Roberts et al., 2016). Second, this division of data also allowed comparison between abundance estimates generated by the modified Horvitz–Thompson estimator for each survey date in the testing dataset and corresponding true counts from ground surveying for that date (Terletzky & Koons, 2016).

The training dataset included 97 unique detections of collared koalas, 44 duplicate detections of collared koalas, 26 radio‐collared koalas that were not detected by the algorithm in RPAS survey footage, and 81 objects falsely misidentified as koalas by the algorithm. The testing dataset included observations of all objects identified as koalas by the algorithm, or the raw count of koalas generated by the automated detection method for each survey. Observations of koalas that were determined to be present through ground surveying but not detected by the algorithm were not included in abundance estimation.

### 2.2 Generalized N‐mixture model

The first approach taken to modeling abundance of koalas involved generalized N‐mixture modeling conducted using the “unmarked” package in R (Chandler, Royle, & King, 2011; Fiske & Chandler, 2011). To reduce the potential impact of duplicate detection on abundance estimates, only detections from flight paths spaced 75 meters apart were entered into this model. This spacing ensured there was no overlap in the images, with the FOV of the drone‐mounted camera essentially forming 150 wide strip transects covering the entirety of each site with a single pass. As a result, there was a total of 9 sites or transects that were visited 5 times between February and August 2018.

For this approach, abundance of koalas was modeled on three distinct processes or formulae. First, the “p‐formula” modeled the impact of distance from observer, in this case the drone, in meters on the probability of detecting koalas (Chandler et al., 2011). To model this, counts of koalas were binned into five distance classes (0–15 m, 15–30 m, 30–45 m, 45–60 m, and 60–75 m) which represented how far away animals were from the position of the drone at the time of detection. A half‐normal detection function, in which detection decreased with increased distance from the drone, a uniform detection function, in which detection did not decrease with increased distance from the drone, and a model in which detection decreased with distance with the effective detection distance depending on percentage forest cover within the transect were investigated as possible p‐formulae (Table 1).

**TABLE 1:** List of covariates investigated for each formula of generalized N‐mixture models of koala abundance at north and south sites at Petrie Mill, Queensland

Formula	Response Variable	Covariates	
		North Site	South Site
P‐formula	Probability of detecting koalas	Distance to observer (m), forest cover (%)	Distance to observer (m), forest cover (%)
Phi‐formula	Probability of detecting koalas	Ambient temperature (℃), wind speed (km/hr)	Ambient temperature (℃), wind speed (km/hr)
Lambda formula	Abundance of koalas	Forest cover (%), Grass cover (%), Road cover (%)	Forest cover (%), Grass cover (%), Water cover (%)

The second formula of the generalized n‐mixture models was the phi‐formula, which modeled the impact of covariates that differed between visits, in this case changing weather conditions, on probability of detecting koalas (Chandler et al., 2011). Possible covariates included ambient temperature and wind speed at the time of survey as increases in both of these have shown to negatively affect image quality for thermal and RPAS‐derived imagery and therefore lower probability of detection (Table 1) (Chretien et al., 2016; Seymour et al., 2017).

The final formula was the lambda formula, which modeled the underlying abundance of koalas depending on differences in habitat structure between transects (Chandler et al., 2011). Percentage cover of different land‐use classes including forest, grass, road (north site only), and water (south site only) was investigated as possible habitat structure covariates for this formula (Table 1). These covariates were selected as forested areas were more likely to be suitable habitat for koalas compared to cleared grass‐covered areas and water, and koalas have been shown to be more likely to be present further from road edges (McAlpine et al., 2006). For all three formulae, the response variable was modeled with a negative binomial error distribution.

### 2.3 Modified Horvitz–Thompson estimator

Separate models for probability of detecting koalas, probability of false detection, and probability duplicate detection were constructed for this approach to modeling koala abundance. A total of 123 observations of koalas that were successfully detected or missed by the algorithm in RPAS survey images were used to model probability of detecting koalas ($p_{i}$) as:

$$Y_{i}∼\text{Bernoulli}\left(p_{i}\right)=\log\left(\frac{p_{i}}{1-p_{i}}\right)=\beta_{0}+\sum_{j=1}^{J}\beta_{j}x_{i,j}$$

where $Y_{i}$ = 1 for successfully detected koalas, $Y_{i}$= 0 for missed koalas, $\beta_{0}$ is the intercept, and $\beta_{j}$ is the change in $Y_{i}$ for every unit change in covariate $x_{i,j}$. Duplicate and false detections were not included in development of this model.

As shown in Table 2, covariates for this model included three factors that have been suggested to negatively affect the quality of thermal images captured by drones which could impact on likelihood of detecting koalas: ambient temperature, wind speed at the time of surveying, and distance of the detected objects to the habitat edge (Chretien et al., 2016; Christie et al., 2016; Seymour et al., 2017). Higher values for distance to habitat edge indicated objects were further from the edge and closer to the forest core. This was included as a possible covariate because animals situated closer to forest cores, where there is typically more overhead obstruction, have been found to be less likely to be detected in RPAS surveys (Chretien et al., 2016; Witczuk, Pagacz, Zmarz, & Cypel, 2018). Ambient temperature and wind speed were included because the quality of images can be negatively affected when either is high which can reduce probability of detection (Witczuk et al., 2018).

**TABLE 2:** List of covariates investigated for inclusion in models of probability of overall detection, false detection, and duplicate detection for koalas automatically identified in RPAS‐derived thermal imagery

Covariate	Unit of Measurement	Probability of Detection Model	False Detection Model	Duplicate Detection Model
Ambient temperature	Degrees Celsius(℃)	Yes	Yes	No
Wind speed	Kilometers per hour (km/hr)	Yes	Yes	No
Distance to habitat edge	Meters (m)	Yes	Yes	No
Distance to nearest detection	Meters (m)	No	No	Yes
Time since previous detection	Seconds (sec)	No	No	Yes

To investigate probability of false detection observations of koalas that were not detected by the algorithm was omitted from model development. Using the remaining 221 observations, the probability that koalas detected by the algorithm were false ($f_{i}$) was modeled as:

$$W_{i}∼\text{Bernoulli}\left(f_{i}\right)=\log\left(\frac{f_{i}}{1-f_{i}}\right)=\gamma_{0}+\sum_{k=1}^{K}\gamma_{k}a_{i,k}$$

where $W_{i}$ = 1 for an object misidentified as a koala and $W_{i}$ = 0 for a verified true detection of a koala, $\gamma_{0}$ is the intercept, and $\gamma_{k}$ is the change in $W_{i}$ for every unit change in covariate $a_{i,k}$. Observation of koalas that were missed was not included in construction of this model. The possible covariates explored for this model were the same as for the model of overall probability of detection (Table 2). The main reason for this was that previous studies have shown as probability of detection for automated wildlife counting methods increases, false detection typically increases; therefore, it is likely variables affecting probability of detection would also influence false detection rate (Kellenberger et al., 2018; Longmore et al., 2017; Rey et al., 2017). Furthermore, previous surveys of koala distribution suggest they are more likely to be found closer to forest cores, so animals detected closer to habitat edges are more likely to be false detections (Ellis, Rhind, Smith, & Lunney, 2017; Januchowski et al., 2008).

To develop models of duplicate detection, only observations that were successfully detected by the algorithm and verified to be koalas were included (*n* = 140); false detections and koalas that were missed in RPAS surveying were not included. The probability that koalas detected by the algorithm were duplicates of previously detected individuals ($d_{i})$ was modeled as:

$$Z_{i}∼\text{Bernoulli}\left(d_{i}\right)=\log\left(\frac{d_{i}}{1-d_{i}}\right)=\kappa_{0}+\sum_{m=1}^{M}\kappa_{m}b_{i,m}$$

where $Z_{i}$ = 1 for detections of koalas that had been successfully detected previously in a survey and $Z_{i}$ = 0 for the first detection of a unique koala,$\kappa_{0}$ is the intercept, and $\kappa_{m}$ is the change in $Z_{i}$ for every unit change in covariate $b_{i,m}$. Distance to nearest detection and time elapsed since the previous detection were investigated as potential covariates (Table 2). These covariates were intended to capture the effect of the overlap in FOV of the RPAS during surveys, as detections that were closer to other detections in time and space were more likely to be duplicates of the same animal viewed from a different angle by the RPAS sensor (Potvin & Breton, 2005; Terletzky & Koons, 2016; Table 2).

Model selection for probability of detection, false detection, and duplicate detection occurred in a stepwise manner. First univariate GLMs were constructed and ranked along with their null models according to *p*‐value, reduction in residual deviance yielded by each model, and Akaike information criteria (Burnham & Anderson, 2002; Posada & Buckley, 2004). All possible combinations of covariates found to explain a significant amount of variance in each response variable were then used to construct multivariate GLMs and ranked against the top‐performing univariate model using the same criterion. Multivariate generalized additive models (GAMs) were also investigated and assessed by AIC ranking. Generalized linear mixed‐effects models (GLMMs) were also explored incorporating random effects of the survey site, but they produced a singular fit given the available data. The percentage of variance explained by the top‐performing model for overall probability of detection, false detection, and duplicate detection was determined by calculating the pseudo R‐squared (McFadden, 1974). All model selection and analysis were completed using R statistical software (R Core Team, 2018).

Estimates of the abundance of koalas at north and south Petrie Mill, Queensland, during each survey were made using a Horvitz–Thompson (H‐T) (Steinhorst & Samuel, 1989; Williams, Nichols, & Conroy, 2002) estimator modified to include duplicate detections based on corrections by Marques et al. (2009) and a novel modification to include the influence of false detection rate. The modified H‐T estimator allowed calculation of the estimated abundance of koalas present during each survey (N) using the equation:

$$N=\sum_{i=1}^{C}\frac{I_{i}*\left(1-\left(\left({\hat{d}}_{i}+\hat{f}i\right)\right)\right)}{{\hat{p}}_{i}}$$

where *C* is the number of objects in thermal images from RPAS surveys that were detected and identified as koalas by the algorithm, *I_i_* is an indicator variable with a value of one for each object identified by the algorithm as a koala, *d_i_* is the estimated probability of detected object *i* being a duplicate, *f_i_* is the estimated probability of detected object *i* being a false detection, and *p_i_* is the estimated probability of detection for individual *i*.

95% confidence intervals around the abundance estimates were created by first predicting the fitted values of *p_i_*, *f_i_*, and *d_i_* plus or minus 1.96 times the standard error (representing the 97.5th quantile of the standard normal distribution) on the link scale, and then using the inverse of the link function to map the fitted values, upper and lower limits back to the response scale. The upper confidence limit of estimated abundance (*N*) could then be calculated by including the lower confidence limits of *p_i_*, *f_i_*, and *d_i_* into the modified H‐T estimator as *N* increases when the chance that detected objects are false or duplicates is low and the likelihood that all koalas were successfully detected in the survey is low (Marques et al., 2009; Steinhorst & Samuel, 1989; Terletzky & Koons, 2016; Williams et al., 2002). Conversely, the lower confidence limit of *N* could be calculated as using the upper confidence limits of *p_i_*, *f_i_*, and *d_i_* as estimated abundance increases when the probability that detected objects were falsely identified as koalas or duplicates is high and the likelihood that all koalas present were successfully detected is high (Marques et al., 2009; Steinhorst & Samuel, 1989; Terletzky & Koons, 2016; Williams et al., 2002). The proportion of surveys for which the true count verified by ground surveying was captured within the 95% confidence intervals was used to assess the accuracy of modified H‐T estimator (Clement, O’Keefe, & Walters, 2015; Gerber, Ivan, & Burnham, 2014; Hickey & Sollman, 2018; Terletzky & Koons, 2016).

## 3 RESULTS

### 3.1 Generalized N‐mixture model

At both sites, the p‐formula model with the lowest AIC was the uniform detection function model, suggesting detection probability did not meaningfully decline with increased distance from the drone (Table 3).

**TABLE 3:** Ranking of p‐formula models for impact of distance from RPAS platform on probability of detecting koalas at north and south sites in Petrie, Queensland

Model	AIC	
	North Site	South Site
Uniform Detection	202.62	216.95
Half‐normal Detection	204.62	218.95
Half‐normal Detection + Forest Cover (%)	206.62	220.95

Temperature did not significantly impact detection of koalas at the north (*p* = .459) or south (*p* = .518) site. Similarly, wind speed did not significantly impact detection or koalas at either site with *p* = .265 at the north site and *p* = .283 at the south site. Adding these covariates to the phi‐formula model also increased AIC compared to a uniform detection function model, and therefore, the null model was retained for the phi‐formula (Table 4).

**TABLE 4:** Ranking of phi‐formula models for impact of covariates on variability of probability of detection between surveys

Model	AIC	
	North Site	South Site
Uniform Detection (Null)	202.62	215.24
Wind Speed	203.24	216.00
Temperature	204.04	216.82
Temperature + Wind Speed	205.24	217.50

The model that provided the best fit at the north site was a model with a uniform detection function p‐formula, null phi‐formula, and percentage of road cover and percentage of grass cover as lambda covariates (Table 5). Percentage of road cover (*β*_percentage road cover_ = −0.0985, *SE* = 0.0330, *p* = .0397) and percentage of grass cover (*β*_percentage grass cover_ = −0.0308, *SE* = 0.0129, *p* = .017) were found to have significant negative impact on the underlying abundance of koalas.

**TABLE 5:** Top‐performing lambda‐formula models of underlying abundance of koalas at North Petrie Mill, Queensland, compared to uniform detection function null model

Model	AIC
Road Cover (%) + Grass Cover (%)	198.10
Road Cover (%) + Forest Cover (%)	198.11
Forest Cover (%) + Grass Cover (%)	198.13
Road Cover (%)	200.30
Uniform Detection (Null)	202.62
Forest Cover (%)	203.71
Grass Cover Percentage (%)	204.55

For the south site, a model with the same p‐formula and phi‐formula but with percentage forest cover and water cover was found to have the best fit (Table 6). Forest cover (*β*_percentage forest cover_ = −0.0510, *p* = .024) and water cover (*β*_percentage water cover_ = −0.0255, *p* = .018) both had a significant negative impact on the underlying abundance of koalas at the site. Based on parametric bootstrapping with 500 simulations, the Freeman–Tukey p‐values for the north site model (*p* = .517) and south site model (*p* = .407) were both over 0.10 indicating adequate model fit.

**TABLE 6:** Top‐performing lambda‐formula models of underlying abundance of koalas at South Petrie Mill, Queensland, compared to uniform detection function null model

Model	AIC
Forest Cover (%) + Water Cover (%)	215.31
Forest Cover (%) + Grass Cover (%)	215.32
Grass Cover (%) + Water Cover (%)	215.34
Uniform Detection	216.95

### 3.2 Modified Horvitz–Thompson estimator

Ambient temperature and distance of koalas from habitat edges were both found to make significant contributions to variation in overall probability of detection (Table 7). Wind speed did not account for a significant amount of variance in probability of detection for koalas (*p* = .1782). Ambient temperature was shown to negatively impact probability of detection, with objects less likely to be detected during surveys when the temperature was higher (*β*_Temperature_ = −0.1879, *SE* = 0.0550). There was also a negative relationship between distance to habitat edge and probability of detection in which objects that were further from habitat edges and closer to forest cores were less likely to be detected (*β*_Distance to habitat edge_ = −0.0233, *SE* = 0.0076).

**TABLE 7:** Top‐performing models of probability of detection for koalas automatically identified in RPAS‐derived thermal imaging from Petrie, Queensland, in 2018

Model Type	Covariates	Null deviance	Residual deviance	*p*‐value	AIC
GAM	Temperature, distance to habitat edge	126.88	83.81	.0231, .0412	115.7
GLM	Temperature, distance to habitat edge	126.88	111.00	.0081, .0303	116
GLM	Temperature	126.88	114.67	.0006	118.67
GLM	Distance to habitat edge	126.88	117.13	.0022	121.13
Null model	N/A	126.88	126.88	N/A	128.88

A multivariate GLM with ambient temperature and distance from habitat edge as covariates which explained 13.33% of variance in probability of detecting koalas in RPAS‐derived thermal imagery using automated methods was found to be the top performing model (Table 7). This model was incorporated into the modified Horvitz–Thompson estimator as a GAM with the same covariates did not meaningfully reduce AIC by more than 2 points (Table 7).

Distance to habitat edge was found to significantly affect the probability that a detection was false with detections that were further from the habitat edge found to be less likely to be false (*β*_Distance to habitat edge_ = −0.0324, *SE* = 0.0071, Table 8). Wind speed was also found to have a significant effect with the probability that a detection was false decreasing when wind speed was greater (*β*_Wind speed_ = −0.1925, *SE* = 0.0886). However, the univariate GLM including distance to habitat edge was incorporated into the modified Horvitz–Thompson estimator as it provided the best fit with the lowest AIC compared to the null model was found to explain 9.23% of the variance in likelihood of false detection (Table 8).

**TABLE 8:** Top‐performing models of probability of falsely detecting koalas in RPAS‐derived thermal imaging using automated methods

Model Type	Covariates	Null deviance	Residual deviance	*p*‐value	AIC
GLM	Distance to habitat edge	290.43	267.62	.000005	267.62
GLM	Distance to habitat edge, wind speed	290.43	269.37	.000005, .6120	269.37
GLM	Wind speed	290.43	285.31	.0297	289.31
Null model	N/A	290.43	290.43	N/A	292.43

Distance to nearest detection was found to significantly influence the likelihood of detected objects being duplicates (*p* = .006), while times since previous detection did not (*p* = −.3720). Detections that were closer to other detections in space (*β*_Distance to nearest detection_ = −0.0228, *SE* = 0.0060) were more likely to be duplicates. The univariate GLM of the effect of distance to nearest detection on the likelihood of duplicate detection had an AIC of 160.35 compared to 174.70 for the null model and was found to account for 9.47% of variation in the likelihood of duplicate detection. Therefore, this model was selected for incorporation into the final modified Horvitz–Thompson estimator.

### 3.3 Abundance estimation

The true ground count of radio‐collared koalas was contained within the 95% confidence interval obtained using the modified H‐T estimator for all four surveys in the testing dataset (Table 9). The confidence intervals were found to larger at the north site compared to the south site (Table 9). The estimated abundance of koalas at both the north site (85) and south site (124) based on the generalized N‐mixture modeling method was much higher than the true abundance at each site confirmed by ground surveys (Table 9).

**TABLE 9:** Abundance estimates (±95% confidence intervals) of koalas for surveys of north and south Petrie Mill, Queensland, in 2018 compared to the raw number of detections generated by the algorithm and the true number of koalas present as determined by ground counts

Survey site	Survey date	Raw count	Estimated abundance ± 90% CI (aerial survey method)	Estimated abundance (generalized N‐mixture method)	True count
North Petrie Mill	10 July 2018	41	15(6,27)*	85	15
	24 July 2018	55	23(10,40)*		18
South Petrie Mill	11 July 2018	17	5(2,9)*	124	9
	24 July 2018	25	9(3,15)*		11

Note

True count (verified with telemetry) within the estimated confidence interval (*).

## 4 DISCUSSION

The generalized N‐mixture method overestimated abundance at both sites, likely because false and duplicate detections were not accounted for in the model (Brack et al., 2018; Ficetola et al., 2018). The algorithm described in Corcoran et al. (2019) yielded raw counts with a lower number of false and duplicate detections compared to previous studies on automated wildlife detection (Corcoran et al., 2019; Kellenberger et al., 2018; Rey et al., 2017). Nonetheless, there were still a number of spurious detections that may have led to overestimates of abundance.

In contrast, the relatively simple modified H‐T estimator approach performed well at estimating the abundance of koalas, with the true number of koalas contained within the 95% confidence intervals in 4 of the 4 surveys. This was due to the modified H‐T estimator enabling probability of detection error across detected koalas to cancel out the combined effects of false and duplicate detections (Marques et al., 2009; Terletzky & Koons, 2016).

Originally developed for abundance estimation from fixed‐wing aircraft, this is apparently the first time that the H‐T estimator has been adapted for use with automated ID data collected from a RPAS survey and extended to include the likelihood that detections may be false, misidentified objects rather than duplicates detections of target animals. The performance of this approach seems promising even when compared to many previous attempts to model abundance from conventional aerial surveys that accounted for probability of detection and duplicate detection. In these surveys, the true count was only found within 33% and 38% of surveys, with the majority of counts inflated due to very low probability of detection (Potvin & Breton, 2005; Terletzky & Koons, 2016). Other models that only accounted for probability of detection found 50%–83% of abundance estimates were significantly below the true ground counts (Lee & Bond, 2016). The results also demonstrate an improvement upon commonly used methods for koala abundance estimation as conventional koala ground surveys typically result in their numbers being underestimated by up to 25% (Dique et al., 2003).

Another advantage of the modified H‐T estimator method was that it did not generate any abundance estimates where the true count was below the lower confidence limit. This is important as overestimating the abundance of threatened species, such as koalas, has been shown to lead to poor decision‐making based on the false impression that a population was stable or recovering (Ficetola et al., 2018; Siddig, Ellison, & Jackson, 2015). It also did not rely on multiple visits to survey sites to achieve reliable estimates and therefore may be better suited to monitoring species for which population data are needed to aid management urgently, or for which resource to conduct monitoring is scarce (Scheele et al., 2018).

As predicted, increased ambient temperature and increased distance of animals to habitat edges negatively impacted the probability of detection, likely due to the decreased contrast between koalas and their background, and potentially greater obstruction between the sensor and the target (Chretien et al., 2016; Hodgson et al., 2016; Seymour et al., 2017). These covariates are likely to play an important role in determining probability of detection for all wildlife surveys using RPAS and thermal imaging, which increase the potential applicability for the modified H‐T estimator approach to a wider range of survey sites and situations, where abundance is unknown and the capacity to mark and ground truth individuals is limited (Chretien et al., 2016; Kellenberger et al., 2018; Seymour et al., 2017). Estimating reasonable values of the covariates for each application would be required unlike using N‐mixture methods, but this trade‐off may be beneficial for species requiring very urgent monitoring. Further collection of data on the types of error in surveys of other species and in different habitat types could widen the applicability of the modeling method (Brack et al., 2018; Chretien et al., 2016; Hodgson et al., 2016; Seymour et al., 2017)

Distance to the nearest detection was a significant predictor of duplicate detection error, as expected, suggesting that the overlap in FOV of the RPAS during survey can lead to accidental double‐counts of the same animal when viewed from different angles (Baxter & Hamilton, 2018; Brack et al., 2018; Denes et al., 2015; Riddle et al., 2010). However, the modified H‐T estimator was able to account for this in most instances, suggesting there is potential for this approach to be used to reliably estimate population size of many species. Further investigation into how RPAS FOV overlap contributes to detection error in surveys of more mobile species is recommended in order to ensure accurate abundance estimates can be made for a wider range of wildlife (Terletzky & Koons, 2016; Witczuk et al., 2018). The existing tracking method used by the algorithm to generate detections could be also be modified based on the parameter estimate for distance to nearest detection to eliminate more duplicate detections at the initial detection stage and reduce the likelihood of inaccurately estimating abundance (Corcoran et al., 2019).

Overall, the results of this study suggest that current N‐mixture modeling techniques may lead to substantial overestimates of abundance of wildlife in RPAS surveys with automated detection (Kellenberger et al., 2018; Longmore et al., 2017; Rey et al., 2017). If these approaches were modified to account for the impact of false and duplicate detections as has been previously suggested (DiRenzio et al., 2019; Kidwai, Jimenez, Louw, Nel, & Marshal, 2019), N‐mixture models may become more useful in conjunction with this particular kind of data, although a satisfying solution to this problem is yet to emerge. Alternatively, the modified H‐T estimator approach developed here may be a promising way forward, as it yielded substantially more accurate estimates than generalized N‐mixture modeling (Dique et al., 2003; Lee & Bond, 2016; Potvin & Breton, 2005; Terletzky & Koons, 2016). This suggests that with auxiliary data collected to determine detection probabilities, counts of wildlife detected using automated methods in RPAS‐derived thermal imaging could be integrated into wildlife monitoring plans in order to efficiently and reliably estimate abundance of cryptic and widespread species (Barker et al., 2018; Brack et al., 2018; Link et al. 2018). While no survey method is universally applicable and RPAS surveys cannot be used in all cases, habitats, or weather conditions, the management of koalas and other threatened species that are difficult to survey through conventional ground or aerial methods could potentially benefit from the improved understanding of their population numbers that could be gained through this powerful new modeling approach (Adams‐Hosking et al., 2016; Scheele et al., 2018).

## CONFLICT OF INTEREST

The authors declare they have no conflicts of interest.

## AUTHOR CONTRIBUTION

**Evangeline Corcoran:** Data curation (lead); Formal analysis (lead); Methodology (lead); Validation (lead); Writing‐original draft (lead); Writing‐review & editing (equal). **Simon Denman:** Conceptualization (supporting); Data curation (supporting); Formal analysis (supporting); Supervision (supporting); Writing‐review & editing (equal). **Grant Hamilton:** Conceptualization (lead); Formal analysis (supporting); Funding acquisition (lead); Investigation (supporting); Methodology (supporting); Project administration (lead); Supervision (lead); Writing‐review & editing (equal).

## Data Availability

Datasets used in model development and testing in this study are available from the Zenodo repository https://doi.org/10.5281/zenodo.3889324.
